# Preserving Fossilized Soft Tissues: Advancing Proteomics and Unveiling the Evolutionary History of Cancer in Dinosaurs

**DOI:** 10.3390/biology14040370

**Published:** 2025-04-03

**Authors:** Pramodh Chitral Chandrasinghe, Biancastella Cereser, Sergio Bertazzo, Zoltán Csiki-Sava, Justin Stebbing

**Affiliations:** 1 Department of Surgery and Cancer, Imperial College London, London W12 0NN, UK; pramodh@kln.ac.lk (P.C.C.);; 2 Department of Surgery, Faculty of Medicine, University of Kelaniya, Kelaniya 11010, Sri Lanka; 3 Department of Medical Physics and Biomedical Engineering, University College London, London WC1E 6BT, UK; s.bertazzo@ucl.ac.uk; 4 Faculty of Geology and Geophysics, University of Bucharest, 010041 Bucharest, Romania; zoltan.csiki@g.unibuc.ro; 5 Institute of Geography and Earth Sciences, ELTE Eötvös Loránd University, 1117 Budapest, Hungary; 6 School of Life Sciences, Anglia Ruskin University, Cambridge CB1 1PT, UK

**Keywords:** paleopathology, paleoproteomics, fossils, evolutionary biology of cancers

## Abstract

Understanding how life-history strategies influence cancer susceptibility in dinosaurs requires a molecular-level analysis of preserved soft tissues. While previous research has largely focused on skeletal remains, the discovery of soft tissue structures in fossils, such as *Telmatosaurus transsylvanicus*, highlights the need for a new approach. Paleoproteomics offers a transformative opportunity to analyze ancient proteins, revealing the evolutionary trade-offs between growth, reproduction, and cancer suppression. This study argues that prioritizing fossil collection and soft tissue preservation is crucial, as future advances in molecular techniques will allow deeper insights into disease evolution. By integrating life-history theory with paleopathology, we can better understand the selective pressures that shaped cancer susceptibility in extinct species and identify potential mechanisms of tumor resistance. This commentary highlights the necessity of long-term fossil conservation efforts to support future breakthroughs in evolutionary biology and comparative oncology.

## Simple Summary

This study explores how cancer in dinosaurs can offer insights into the evolutionary history of disease and life-history strategies. By using paleoproteomics to analyze preserved proteins in fossils, researchers are uncovering the molecular markers of disease in ancient species. The discovery of tumors, particularly in hadrosaurs and the recent finding in *Telmatosaurus transsylvanicus*, highlights the potential of studying cancer in extinct species. The integration of life-history theory with advanced proteomic methods aims to reveal how evolutionary trade-offs influenced cancer susceptibility and may uncover new mechanisms of cancer suppression. The research emphasizes the importance of preserving fossilized tissues and advancing proteomic techniques to understand the evolution of cancer and improve modern disease prevention.

## 1. Introduction

The study of ancient diseases provides a unique window into evolutionary biology, helping to uncover the fundamental mechanisms of tumorigenesis and tumor suppression. However, our ability to answer these questions is limited by the availability of well-preserved fossils. As paleoproteomic techniques advance, studying fossilized soft tissues will become crucial for reconstructing ancient life-history strategies and understanding disease evolution.

By prioritizing the collection and analysis of fossils with preserved soft tissues, we can ensure that future technological advancements in proteomics will have sufficient material to analyze. The fossils excavated today will provide crucial material for future molecular analyses, enabling deeper investigations into ancient diseases and life-history evolution, providing unprecedented insights into how extinct species regulated cancer susceptibility and evolved mechanisms for disease suppression.

### 1.1. Life-History Theory and Cancer

Life-history theory in evolutionary biology explains how organisms allocate limited resources to growth, reproduction, and survival in response to environmental pressures. These allocations are shaped by trade-offs, where investment in one function (e.g., reproduction) necessarily reduces the resources available for others (e.g., survival or growth) [1]. Understanding these strategies across evolutionary timescales provides valuable insights into health and disease across species. While traditional life-history studies, extensively developed by Stephen Stearns and Derek Roff starting in the late 1970s, initially overlooked cancer [1,2], the discovery of tumors in dinosaurs, particularly in the caudal vertebrae of Late Cretaceous hadrosaurs, offers a unique perspective. Large body size presents a recurring challenge in evolution: how to minimize the risk of cancer in organisms with many cells and extended lifespans [3]. Cancer, viewed through the lens of life-history theory, emerges not only as a disease but as a potential consequence of the trade-offs organisms make in allocating resources [4]. The widespread occurrence of cancer across the tree of life [5] highlights its deep evolutionary roots and its connection to the challenges of multicellularity.

Evidence from the fossil records reveal malignant lesions in extinct species, suggesting that cancer may have exerted selective pressures on ancient populations [3,6]. Several tumors, including metastatic lesions, have been found in dinosaurs, especially in hadrosaur fossils, challenging the idea that cancer was a rare event in these species [7]. These findings suggest that cancer was an ongoing occurrence for large organisms like dinosaurs, much like modern, large, long-lived animals such as elephants and whales. However, while we know that elephants have evolved multiple copies of the crucial tumor suppressor gene TP53 [8], and that a bowhead whale relies on efficient DNA repair mechanisms to maintain genome integrity [9], little is known about how dinosaurs managed cancer risk. Did they possess similar genetic adaptations, or did they rely on entirely different mechanisms shaped by their unique physiology and environment?

To further explore these questions, we must turn to the molecular evidence preserved in fossils. By applying modern analytical proteomic techniques, we can now identify soft tissue remnants, and in the future, we may even recognize preserved proteins or the molecular markers of disease.

### 1.2. The Need for Fossil Collection and Soft Tissue Analysis

Despite increasing evidence of tumors in dinosaurs, our ability to study ancient disease is restricted by the scarcity of well-preserved soft tissues. While DNA degrades rapidly over time [10], proteins are much more stable and can persist for millions of years under favorable conditions [11]. The identification of proteins in fossils provides critical information about an organism’s phenotype, such as skin or feather pigmentation, and, at an appropriate resolution, could potentially offer valuable clues about the mechanisms of ancient diseases [11,12].

However, protein preservation is highly dependent on geological and environmental factors and fossils must be carefully collected and stored in conditions that minimize degradation. By employing non-destructive imaging methods to identify soft tissue remnants, we can systematically build a repository of specimens optimized for future proteomic analyses. This will ensure that we maximize scientific returns from every paleontological discovery.

### 1.3. Cancer in the Fossil Record

The recognition of cancer in fossils has expanded significantly in recent years. Although early studies focused on skeletal abnormalities, advanced imaging and histological techniques have confirmed the presence of tumors in non-avian dinosaurs [7,13,14,15,16,17]. Since the late 1990s, several instances of benign and malignant tumors have been documented in dinosaur fossils, such as hemangiomas, desmoplastic fibromas, osteoblastomas, and metastatic cancer [7,13,14,15,16,17]. These tumors have been particularly prevalent in the caudal vertebrae of *Edmontosaurus*, a phytophagous dinosaur that lived during the Late Cretaceous (73 to 66 million years).

The increasing recognition of ancient cancer underscores the need for molecular approaches to further characterize these pathologies.

One particularly remarkable case is the identification of a benign odontogenic tumor in the jaw of the Late Cretaceous non-hadrosaurid hadrosauroid dinosaur *Telmatosaurus transsylvanicus* from the Hațeg Basin in Romania (specimen LPB (FGGUB) R.1305), which we have reanalyzed here.

## 2. Methodology

### 2.1. Advancing Paleoproteomic Techniques

Paleoproteomics, which involves the analysis of preserved proteins in ancient fossils, is a powerful tool for studying the biology and pathologies of extinct organisms. The detection of proteins in ancient fossils has seen significant advancements, primarily through techniques like mass spectrometry [18], scanning electron microscopy (SEM), and Time-of-Flight Secondary Ion Mass Spectrometry (ToF-SIMS) [19]. These methods have proven particularly effective at identifying preserved proteins in badly preserved tissues, such as fossilized bone, where collagen and keratin fragments are often detected. Mass spectrometry, for example, analyzes the mass-to-charge ratios of peptides, enabling researchers to identify protein residues, while SEM provides high-resolution imaging that allows for the visualization of structural features like erythrocyte-like cells and fibrous structures, suggesting the preservation of soft tissue [20]. Additionally, studies using ToF-SIMS have identified amino acid residues such as hydroxyproline and hydroxylysine, which are markers of collagen preservation [13]. Immunological assays have also been employed to confirm the presence of vertebrate-specific proteins, such as osteocalcin, in dinosaur bones, further supporting the preservation of biological molecules in ancient specimens [21]. Finally, synchrotron infrared microspectroscopy has been used to detect amino acids in bone matrix fibrils, confirming the preservation of proteins [22].

These techniques have not only been used to examine bones but have also been applied to other fossilized tissues. For instance, mass spectrometry and ToF-SIMS were used to successfully detect porphyrin molecules derived from hemoglobin in a blood-engorged mosquito fossil, confirming the preservation of molecular structures [23]. Similarly, SEM, Transmission Electron Microscopy (TEM), and ToF-SIMS were employed to confirm the preservation of melanin in melanosome-like microbodies from an Eocene fish eye, further demonstrating how advanced methods can reveal molecularly preserved pigments and other biological components in ancient fossils [24].

While these methods have successfully detected proteins in fossilized bone and soft tissues, their full potential remains currently unexploited. Expanding research efforts on fossil soft tissue preservation will significantly enhance our ability to study ancient biomolecules.

### 2.2. Our Approach

**Figure 1:**
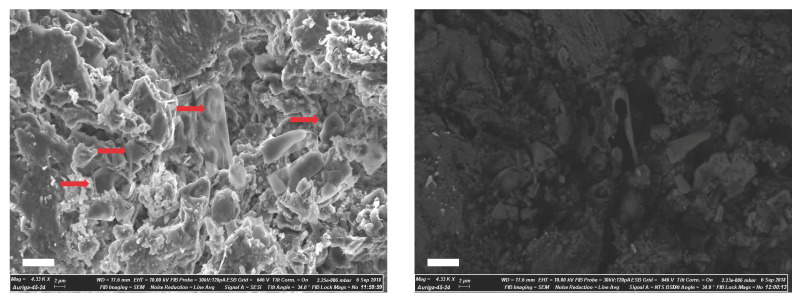
Representative SEM images of fossilized erythrocyte-like structures, highlighting surface topography and density-dependent contrast. (**left panel**) SEM images in secondary electron mode (SES), illustrating the surface morphology of possible erythrocyte-like structures (red arrows) in the pathological *Telmatosaurus transsylvanicus* dentary. (**right panel**) Corresponding backscatter electron images for the same fields of view as (**left**), emphasizing density variations within the structures. All images were acquired at a resolution of 10.00 kV.

We conducted SEM imaging on *Telmatosaurus transsylvanicus* to assess soft tissue preservation within its ameloblastoma lesion (Figure 1). The specimen was kindly provided by Dr. Csiki-Sava from the University of Bucharest; it was previously described in [17] and was originally collected from an outcrop of the Sînpetru Formation along the Sibişel River in the central part of the Hațeg Basin. The specimen is considered to represent a sub-adult individual of *Telmatosaurus transsylvanicus* and is housed at the Laboratory of Paleontology, Faculty of Geology and Geophysics, University of Bucharest. The sample consists of a well-preserved lower dentary with in situ dentition, specifically associated with a neoplastic lesion in the bone. This discovery marked the first reported instance of ameloblastoma in a dinosaur, which was initially identified using non-destructive imaging techniques such as micro-CT scanning and 3D surface imaging [17]. SEM was employed as previously described [20]. The sample was secured to an aluminum sample holder using carbon tape and paste to ensure stability during imaging. It was then coated with 5 nm of carbon using a Turbo-Pumped Thermal Evaporator (model K975X, Quorum Technologies, Laughton, UK), followed by an additional 5 nm layer of chromium using a Sputter Coater (model K575X, Quorum Technologies, Laughton, UK). After coating, the sample was imaged using a Gemini 1525 FEGSEM (Carl Zeiss Microscopy GmbH, Jena, Germany) operating at 10 kV. The SEM was equipped with both an in-lens detector, which recorded secondary electrons, and a backscatter electron detector to capture compositional contrast.

In line with the findings of Bertazzo et al., who identified fibrous patterns consistent with calcified collagen fibers in Late Cretaceous dinosaur fossils [20], we observed low-density structures resembling putative erythrocytes in the fossilized bone and, in particular, within the ameloblastoma lesion (Figure 1). These structures suggest the potential preservation of cellular components within the tumor, further confirming the significant preservation of soft tissues in fossilized specimens. Bertazzo et al.’s previous findings were supported by additional techniques, including focused ion beam (FIB) milling, TOF-SIMS, and electron energy loss spectroscopy (EELS) [20]. This multi-method approach reinforces the interpretation of the erythrocyte-like structures observed via SEM, suggesting that such features, even when detected by SEM alone, are likely genuine, although they may require validation through complementary techniques.

## 3. Discussion

### The Future of Soft Tissue Paleoproteomics

The discovery of preserved cellular structures in fossils, such as *Telmatosaurus transsylvanicus*, presents exciting opportunities for future research in paleopathology and paleoproteomics. Our identification of erythrocyte-like structures within this specimen using SEM stresses the potential for further discoveries of soft tissues and cellular components in ancient fossils. This finding suggests that soft tissues—including cellular structures such as erythrocytes—may be more widespread in the fossil records than previously thought. As paleoproteomic methods advance, the collection and preservation of these specimens will be crucial for investigating prehistoric diseases, including cancer, and their evolutionary trajectories.

Cancer occurrence in dinosaurs challenges the traditional assumptions about life-history trade-offs and raises important questions about how large-bodied species managed cancer risk. A relevant concept in this discussion is Peto’s paradox [25], which postulates that cancer incidence does not scale proportionally with body size or lifespan. Garcês et al. compiled a comprehensive dataset on fossil tumors, revealing that neoplasms have existed for at least 350 million years and are more widespread in the fossil records than previously recognized [26]. Their work highlights the diversity of tumor types and anatomical locations in different dinosaur taxa, reinforcing the idea that cancer was not restricted to specific groups. This growing dataset supports the notion that the mechanisms of tumorigenesis and suppression evolved repeatedly across vertebrate evolution, including in non-avian dinosaurs. Growth strategies in dinosaurs therefore complicate the relationship between body size and cancer susceptibility. A histological analysis of theropod bones has shown that body size evolution was influenced by both growth rate and growth duration, rather than simply slower growth leading to a larger size, which challenges assumptions about the relationship between body size and cancer suppression [27]. Instead, a combination of factors—including metabolic rates, reproductive strategies, and environmental pressures—likely influenced tumor dynamics in these extinct species.

The study of preserved proteins in fossils could help us to understand the paradox. Proteins, particularly those found in calcified tissues like bone, are more stable than DNA and are less susceptible to degradation and contamination [10,28,29]. This makes them ideal candidates for studying ancient diseases, including cancer, in paleontological specimens. In particular, the identification of the proteins involved in cancer-related pathways could reveal whether dinosaurs had oncogenic mutations or tumor-suppressor mechanisms similar to those observed in modern animals. The potential for molecular insights from fossilized soft tissues is particularly exciting. One avenue of research involves examining mutations in cancer-related pathways, such as the MAPK signaling pathway. In modern animals, mutations in this pathway—especially the BRAF V600E mutation—are frequently associated with ameloblastomas, a type of tumor that typically originates in the mandible. This mutation has been documented across multiple species, including humans, dogs, and mice [30,31]. If similar mutations were present in the *Telmatosaurus* tumor, it would suggest that some tumorigenic pathways in dinosaurs were evolutionarily conserved. This would provide deeper insights into the evolutionary dynamics of cancer and highlight the molecular mechanisms that have persisted across vast stretches of evolutionary time.

Beyond paleobiology, studying cancer in dinosaurs has broader implications for modern comparative oncology. Investigating whether large dinosaurs shared specific tumor-suppressor genes with today’s cancer-resistant megafauna could reveal ancient genetic adaptations relevant to contemporary cancer biology. Additionally, exploring the environmental and ecological factors—such as atmospheric oxygen levels and climate conditions—that may have influenced carcinogenesis in the Mesozoic could enhance our understanding of how external factors shape cancer progression at a cellular level.

## 4. Conclusions

The study of ancient diseases through fossilized soft tissues offers an unprecedented opportunity to unravel the evolutionary history of cancer and its relationship with life-history strategies. Dinosaurs, as long-lived, large-bodied organisms, present a compelling case for investigating how species managed cancer susceptibility and resistance over millions of years. The identification of tumors, such as the ameloblastoma in *Telmatosaurus transsylvanicus*, suggests that cancer was not an anomaly but a recurring biological challenge in prehistoric ecosystems.

However, the ability to fully explore these questions depends on the availability of well-preserved fossils containing soft tissues. Unlike skeletal structures alone, soft tissues provide molecular information that can reveal the underlying biological mechanisms of disease. As paleoproteomic techniques advance, these preserved tissues will become crucial in detecting ancient proteins, identifying tumor-suppressor pathways, and understanding how extinct species managed oncogenesis.

Prioritizing fossil collection efforts and improving soft tissue analysis methodologies will ensure that future researchers have access to specimens suitable for cutting-edge molecular investigations. The fossils we study today may not immediately yield all their secrets, but by preserving them under optimal conditions, we prepare for future breakthroughs that could reshape our understanding of cancer evolution.

This research extends beyond paleontology; it has implications for modern comparative oncology. By studying ancient tumor suppression mechanisms, we may uncover biological strategies that could inspire new approaches to cancer treatment in living species. Furthermore, investigating how environmental factors influenced prehistoric cancer incidence could inform our understanding of modern carcinogenesis and its ecological drivers.

To make meaningful progress, an interdisciplinary approach is essential—bridging the expertise of paleontologists, molecular biologists, and evolutionary scientists. Through a collective effort to document, preserve, and analyze fossilized soft tissues, we can unlock the molecular history of ancient diseases and contribute to both evolutionary biology and modern medicine.

## Data Availability

The original contributions presented in this study are included in the article. Further inquiries can be directed to the corresponding author(s).
